# Structure of the Catalytic Domain of EZH2 Reveals Conformational Plasticity in Cofactor and Substrate Binding Sites and Explains Oncogenic Mutations

**DOI:** 10.1371/journal.pone.0083737

**Published:** 2013-12-19

**Authors:** Hong Wu, Hong Zeng, Aiping Dong, Fengling Li, Hao He, Guillermo Senisterra, Alma Seitova, Shili Duan, Peter J. Brown, Masoud Vedadi, Cheryl H. Arrowsmith, Matthieu Schapira

**Affiliations:** 1 Structural Genomics Consortium, University of Toronto, Toronto, Ontario, Canada; 2 Department of Pharmacology and Toxicology, University of Toronto, Toronto, Ontario, Canada; 3 Princess Margaret Cancer Centre and Department of Medical Biophysics, University of Toronto, Toronto, Ontario, Canada; University of Dundee, United Kingdom

## Abstract

Polycomb repressive complex 2 (PRC2) is an important regulator of cellular differentiation and cell type identity. Overexpression or activating mutations of EZH2, the catalytic component of the PRC2 complex, are linked to hyper-trimethylation of lysine 27 of histone H3 (H3K27me3) in many cancers. Potent EZH2 inhibitors that reduce levels of H3K27me3 kill mutant lymphoma cells and are efficacious in a mouse xenograft model of malignant rhabdoid tumors. Unlike most SET domain methyltransferases, EZH2 requires PRC2 components, SUZ12 and EED, for activity, but the mechanism by which catalysis is promoted in the PRC2 complex is unknown. We solved the 2.0 Å crystal structure of the EZH2 methyltransferase domain revealing that most of the canonical structural features of SET domain methyltransferase structures are conserved. The site of methyl transfer is in a catalytically competent state, and the structure clarifies the structural mechanism underlying oncogenic hyper-trimethylation of H3K27 in tumors harboring mutations at Y641 or A677. On the other hand, the I-SET and post-SET domains occupy atypical positions relative to the core SET domain resulting in incomplete formation of the cofactor binding site and occlusion of the substrate binding groove. A novel CXC domain N-terminal to the SET domain may contribute to the apparent inactive conformation. We propose that protein interactions within the PRC2 complex modulate the trajectory of the post-SET and I-SET domains of EZH2 in favor of a catalytically competent conformation.

## Introduction

Enhancer of zeste homolog 2 (EZH2) is the catalytic component of polycomb repressive complex 2 (PRC2), an epigenetic regulator of stem cell pluripotency, and expression of tissue-specific genes involved in cellular differentiation and developmental programs [1,2,3,4,5].. EZH2 carries out a key function of the PRC2 complex, namely the sequential mono-, di- and trimethylation of Lysine 27 of histone H3 (H3K27) within chromatin. H3K27me3 is an epigenetic mark associated with transcriptional repression and contributes to repression of developmental genes, thereby participating in the maintenance of stem cell pluripotency. EZH2 contains a C-terminal SET-domain, a conserved feature of histone lysine methyltransferases [6,7,8]. However, EZH2 by itself does not demonstrate any methyltransferase activity. The catalytic activity of EZH2 requires the presence of at least two other members of the PRC2 complex, namely embryonic ectoderm development (EED) and suppressor of zeste 12 (SUZ12) [9,10,11]. Two additional subunits, the histone-binding protein RBBP4 and the Zinc finger protein AEBP2 together further stimulate EZH2 enzymatic activity [12]. 

Overexpression of EZH2 has been found in a number of human cancers [13,14]. Increased expression levels of EZH2 and increased levels of H3K27me3 are related to tumor development and are associated with poor clinical outcome [15,16,17,18,19]. Elevated expression of EZH2 has also been identified as a marker for breast cancer initiating cells, possibly reflecting its role in maintaining “stemness” [15,20]. In recent studies, missense mutations in EZH2 have been identified in a subset of lymphomas [21,22,23,24,25,26]. Mutations of Y641 (residue numbering according to GeneBank isoform C; Uniprot isoform 1) can increase trimethylase activity of EZH2, thereby leading to elevated global levels of the H3K27me3 mark in mutant cells retaining one wild type allele. In addition, another mutation within the EZH2 SET domain, A677G, has also been identified in Lymphomas and shown to have increased trimethylation efficiency. Together this data suggests a causative role for elevated catalytic activity of EZH2 in the development of cancer. 

Because of the important role of EZH2 in tumorigenesis, much effort has been put into discovery of inhibitors of EZH2 catalytic activity. Recently, several groups reported novel inhibitors of EZH2 [27,28,29,30,31,32]. These compounds are selective for EZH2 over other SET domain methyltransferases, are able to inhibit catalytic activity of both wild-type and lymphoma-associated mutants of EZH2 and reduce the cellular level of H3K27me3. They also showed antiproliferative activity in a subset of lymphoma cell lines carrying EZH2 mutants, while the effects on cells carrying wild-type EZH2 were minimal. Finally, EZH2 inhibition induced regression of pediatric rhabdoid tumors, which are almost always dependant on EZH2 activity [28]. Enzyme kinetic studies indicate that these compounds compete with the co-factor S-adenosyl-methionine (SAM), suggesting they bind to the SAM-binding pocket in the SET-domain of EZH2. Stapled peptides that disrupt the EZH2-EED interaction also have antiproliferative activity in an MLL-rearranged leukemia cell line [33]. These results support the potential of EZH2 as a therapeutic target especially in cancers with overexpressed EZH2 or activating mutations. 

To better understand the molecular mechanism of EZH2 function, we solved the crystal structure of the C-terminal region of EZH2 containing a novel CXC domain and the catalytic I-SET, SET, and post-SET domains. Although the majority of canonical SET domain characteristics were observed, our structure reveals significant differences between the arrangement of the I-SET and post-SET regions compared to that of other SET-domain containing proteins. An unusual conformation of the post-SET domain suggests a potential mechanism of activation of EZH2 by the other core PRC2 subunits. Our structure also provides insight into the gain-of-function mutations in lymphomas.

## Materials and Methods

### Cloning, expression and purification

A DNA fragment encoding residues 520-746 of human EZH2 (Uniprot isoform 1) was sub-cloned from cDNA (GenBank BC010858) into the baculovirus expression vector pFBOH-LIC (GenBank EF456740).  The pFBOH-LIC N-terminal Hexa-His tag was removed by inserting the gene at an upstream Nco1 restriction site and a C-terminal Hexa-His tag introduced by PCR. The resulting expressed protein product is expected to have the AA sequence: MYQPCDHPRQPCDSSCPCVIAQNFCEKFCQCSSECQNRFPGCRCKAQCNTKQCPCYLAVRECDPDLCLTCGAADHWDSKNVSCKNCSIQRGSKKHLLLAPSDVAGWGIFIKDPVQKNEFISEYCGEIISQDEADRRGKVYDKYMCSFLFNLNNDFVVDATRKGNKIRFANHSVNPNCYAKVMMVNGDHRIGIFAKRAIQTGEELFFDYRYSQADALKYVGIEREMEIPHHHHHH. The protein was expressed in Sf9 cells (Invitrogen). The harvested cells were resuspended in lysis buffer containing 20 mM Tris-HCl, pH 8.0, 500 mM NaCl, 5 mM imidazol, 2 mM β-mercaptoethanol, 5% glycerol, 0.6% NP-40, protease inhibitor cocktail (Roche), 3000 U of benzonase (Novagen). Cells were lysed by brief sonication. The clarified lysate was loaded onto a 2 mL TALON column (Clonetech). The column was washed with 50 column volumes of 20 mM Tris-HCl buffer, pH 8.0, containing 500 mM NaCl, 5% glycerol and 5 mM imidazole. The bound protein was eluted with elution buffer containing 20 mM Tris-HCl, pH 8.0, 500 mM NaCl, 5% glycerol, 250 mM imidazole. The eluted protein was further purified to homogeneity on a Superdex200 column (GE Healthcare), equilibrated with 20 mM Tris-HCl buffer, pH 8.0, and 500 mM NaCl.

### Crystallization

Purified EZH2 (10 mg/mL) was mixed with SAM at 1:10 molar ratio of protein:SAM and crystallized using sitting drop vapor diffusion method at 20 °C by mixing 1 µl of the protein solution with 1 µl of the reservoir solution containing 20% PEG 3,350, 0.1 M HEPES, pH 7.5, 0.2 M Li SO4. The crystals were frozen in liquid nitrogen using 10% ethylene glycol as cryo protectant.

### Data Collection and Structure Determination

An initial 2.24 Å diffraction data was collected at the Canadian Light Source (CLS) beamline CMCF 08ID-1 at the Zn absorption edge and used to solve the structure of EZH2 by the single-wavelength anomalous dispersion (SAD) phasing method. A second data set (2.0 Å) collected at beam line 19ID of Advanced Photon Source (APS), Argonne National Laboratory was used to further refine of the structure. All data sets collected at a temperature of 100K and were processed using the HKL-3000 suite [34]. REFMAC [35] was used for structure refinement. The graphics program COOT [36] was used for model building and visualization. MOLPROBITY[37] was used for structure validation and statistics.

### Activity Assays

Methyltransferase activity assays for trimeric EZH2 complex (EZH2-EED-SUZ12) and EZH2 (520-746) were performed by monitoring the incorporation of ^3^H-labeled methyl group into a peptide corresponding to residues 21 to 44 of histone H3 [H3(21-44)] using Scintillation Proximity Assay (SPA). The enzymatic reactions were conducted in triplicate at 23 °C with 0.5 hour incubation of 20 µl reaction mixture in 20 mM Tris-HCl, pH 8.0 (5 mM DTT, 0.01% Triton X-100, 2 µM ^3^H-SAM (Cat.# NET155V250UC; Perkin Elmer; www.perkinelmer.com), 8 µM cold-SAM, 2 µM H3(21-44) peptide) and various concentrations of enzymes. Enzymatic reactions were stopped by adding 7.5 M Guanidine hydrochloride followed by 180 µl of buffer (20 mM Tris-HCl, pH 8.0), mixing and then transferring to a 96-well FlashPlate (Cat.# SMP103; Perkin Elmer; www.perkinelmer.com). After mixing, the reaction mixtures in Flash-plates were incubated for 2 hour and the CPM were measured using Topcount plate reader (Perkin Elmer, www.perkinelmer.com). 

The kinetic parameters for trimeric EZH2 complex were determined using 20 nM of EZH2 at a fixed concentration of peptide (5 µM) and varying concentrations of SAM (up to 20 μM), or at a fixed concentration of SAM (10 µM) and varying concentrations of peptide (up to 5 μM) and therefore kinetic parameters are considered apparent values at the above mentioned conditions. Assays were performed in 20 mM Tris-HCl pH 8, 0.01% Triton X-100, 5 mM DTT. The reaction mixtures were incubated for 30 min at 23 °C. To stop the reactions, 7.5 M Guanidine hydrochloride was added and mixed. A total of 10 µl of the reaction mixture was spotted onto SAM2® Biotin Capture Membrane (cat# V2861, Promega) and placed at room temperature for 5 minutes. Membranes were washed with 2 M NaCl and deionized water at least twice each and were dried. Scintillation liquid was added and counts per minute (CPM) were measured. Experiments were performed in triplicate. 

### ITC measurements

Purified EZH2 (520-746) was dialyzed for binding assay with SAM and peptide in 50 mM Tris-HCl buffer, pH 8.0 and 250 or 500 mM NaCl respectively. Solutions of 1 mM SAM or 0.62 mM histone peptide H3 (residues 21-44) in dialysis buffer were injected into the sample cell containing approximately 1.4 ml of 0.05 mM protein solution. ITC titrations were performed on a VP-ITC Micro Calorimeter from GE Healthcare at 25 °C by using 10 μl injections with a total of 25 injections.

### Structural analysis

Analysis of crystallographic structures was conducted with ICM (Molsoft, san Diego). “Distances” between the two edges of the substrate binding groove were calculated as the distance between the backbone carbonyl of the catalytic tyrosine, at the C-terminal extremity of the SET domain, and the backbone nitrogen of the I-SET domain which forms a conserved hydrogen bond with the backbone carbonyl of the substrate lysine in all available ternary SET domain methyltransferase structures[38]. These two atoms are themselves linked by a hydrogen bond in the EZH2 structure.

## Results

### EZH2 has a canonical SET domain scaffold with atypical subdomain features

**Figure 1:**
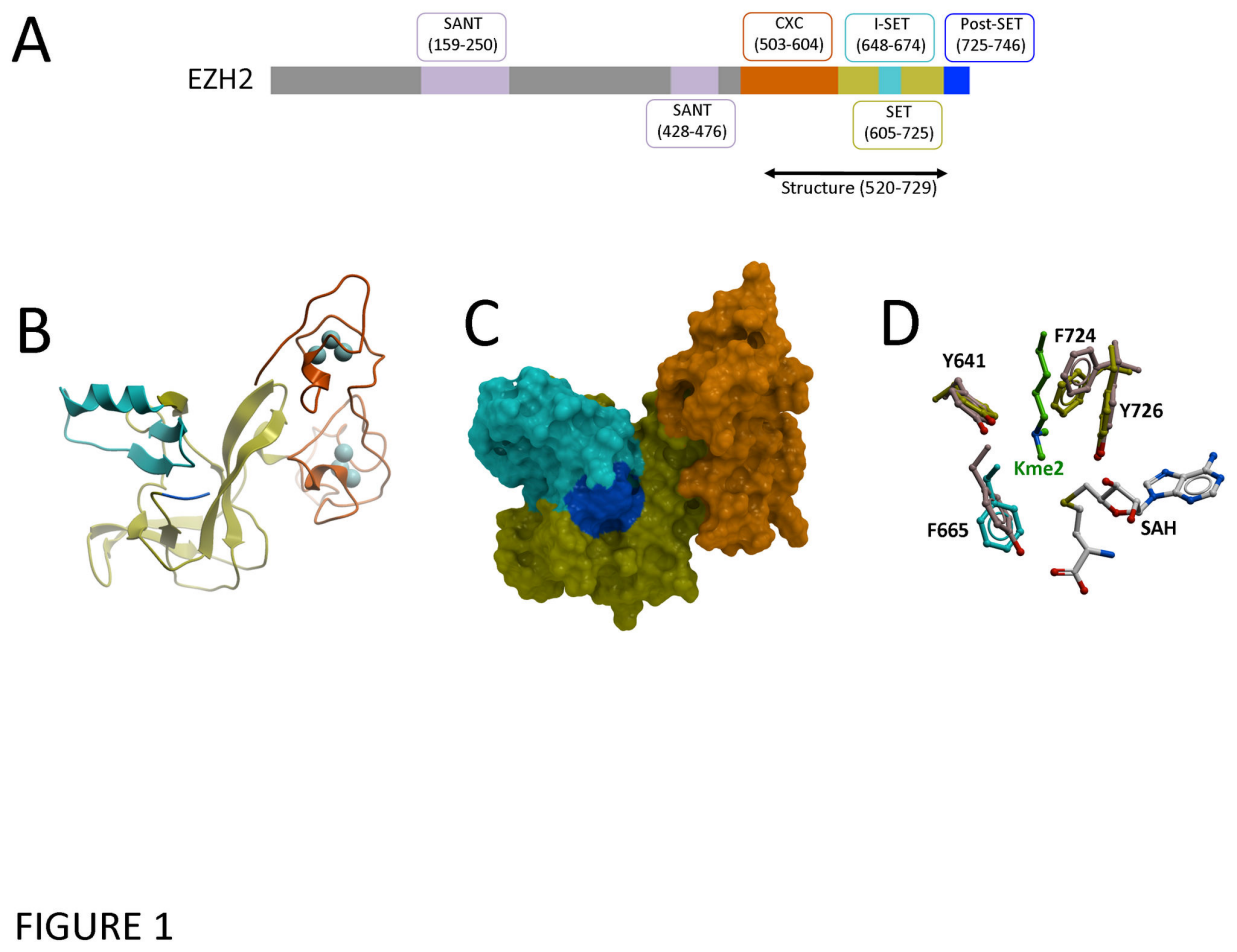
EZH2 adopts the canonical fold of SET domain methyltransferases. (A) Linear domain architecture of EZH2 showing the crystallized construct. Residue numbers according to GenBank isoform C (Uniprot isoform 1). (B) The catalytic SET domain (yellow) is folded as previously described for other histone methyltransferases such as EHMT1/GLP and MLL, but the post-SET domain is largely unresolved and its first five residues (blue) are oriented away from its expected position. The unique CXC domain adopts a novel conformation including two clusters of three Zn ions (light blue spheres). (C) A mesh representation of the EZH2 structure in the same orientation. The cofactor is expected to bind at the junction of the SET, post-SET and I-SET (cyan) domains. (D) Residues forming the substrate lysine-binding channel in EHMT1/GLP (beige – PDB code 2RFI) are structurally conserved in EZH2 (color coding as in A-C).

We crystallized and solved the 2.0 Å structure of the C-terminus of EZH2 (residues 520-746; Figure 1A) (Table 1, Figure S1). Clear electron density was observed for the CXC, SET, and I-SET domains, and the first 5 residues of the post-SET domain. However, the 17 C-terminal residues including most of the post-SET domain were not observed. The cofactor SAM, present in the crystallization buffer, was also absent from the structure. 

**Table 1:** Crystallography data and refinement statistics.

	EZH2
**PDB Code**	4MI0
**Data collection**	
Space group	P2_1_2_1_2_1_
Cell dimensions	
*a*, *b*, *c* (Å)	45.0, 57.8, 74.5
α,β,γ (°)	90.0,90.0,90.0
Resolution (Å) (highest resolution shell)	50.00-2.00 (.03-2.00)
Measured reflections	62070
Unique reflections	13445
*R*_merge_	8.4(53.9)
I/σI	19.4
Completeness(%)	98.0(94.1)
Redundancy	4.6(4.2)
**Refinement**	
Resolution (Å)	32.06-2.00
No. reflections (test set)	13414(654)
*R*_work/_ *R*_free_ (%)	23.9/19.8
No. atoms	
Protein	1584
Zn	6
Water	64
B-factors (Å^2^)	
Protein	36.2
Zn	27.7
Water	36.9
RMSD	
Bond lengths (Å)	0.010
Bond angles (°)	1.13
Ramachandran plot % residues	
Favored	96.1
Additional allowed	3.9
Generously allowed	0
Disallowed	0

Overall the EZH2 structure displays most of the canonical features of SET domain protein methyltransferases (Figure 1B-D) [38]. First, the SET domain forms a central scaffold harboring the active site and including a characteristic pseudo-knot (Figure 1B). Second, the I-SET domain forms a β-hairpin that typically participates in formation of the substrate binding groove (Figure 1B). Third, a cluster of conserved aromatic residues line the methyl-lysine binding channel (including the catalytic tyrosine Y726) and superimpose well with ternary structures of other SET domain methyltransferases bound to cofactor and substrate (Figure 1D). This high level of structural conservation in the lysine channel/active site allows the identification of key residues that participate in catalysis (see below). Interestingly, the catalytic site of EZH2 is structurally closer to that of the human H3K9 dimethylase EHMT1 than the viral H3K27 trimethylase vSET (Figure S2), which is in agreement with the latter’s distinctly different enzymatic properties [39]. Finally, the putative substrate binding site is electronegative (Figure S3), as expected for interactions with the highly basic H3 histone tail.

**Figure 2:**
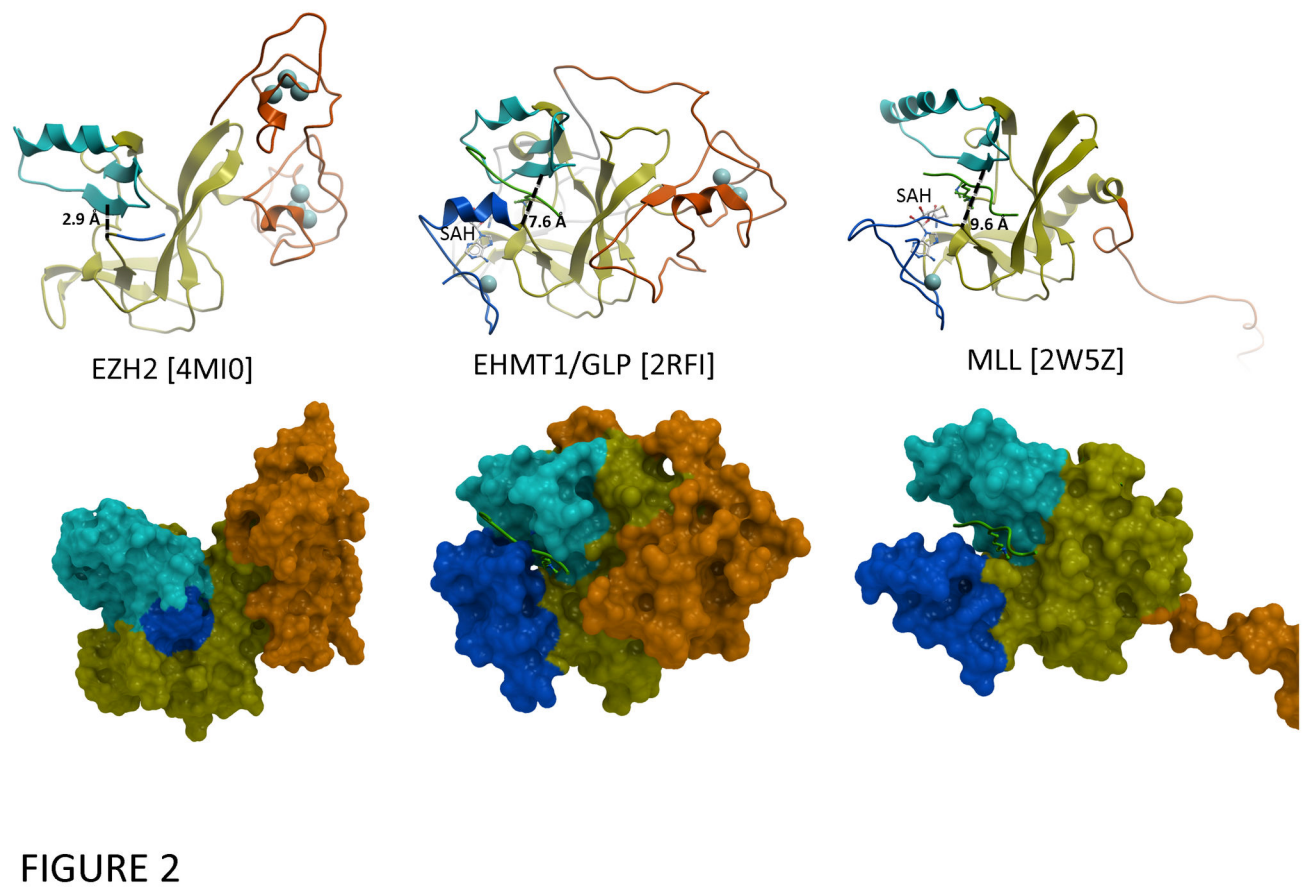
The substrate binding site of EZH2 is occluded. The substrate binding groove is too wide in MLL (right) and too narrow in EZH2 (left), compared with the catalytically competent state observed in the EHMT1/GLP ternary complex (center). Color-coding as in Figure 1.

**Figure 3:**
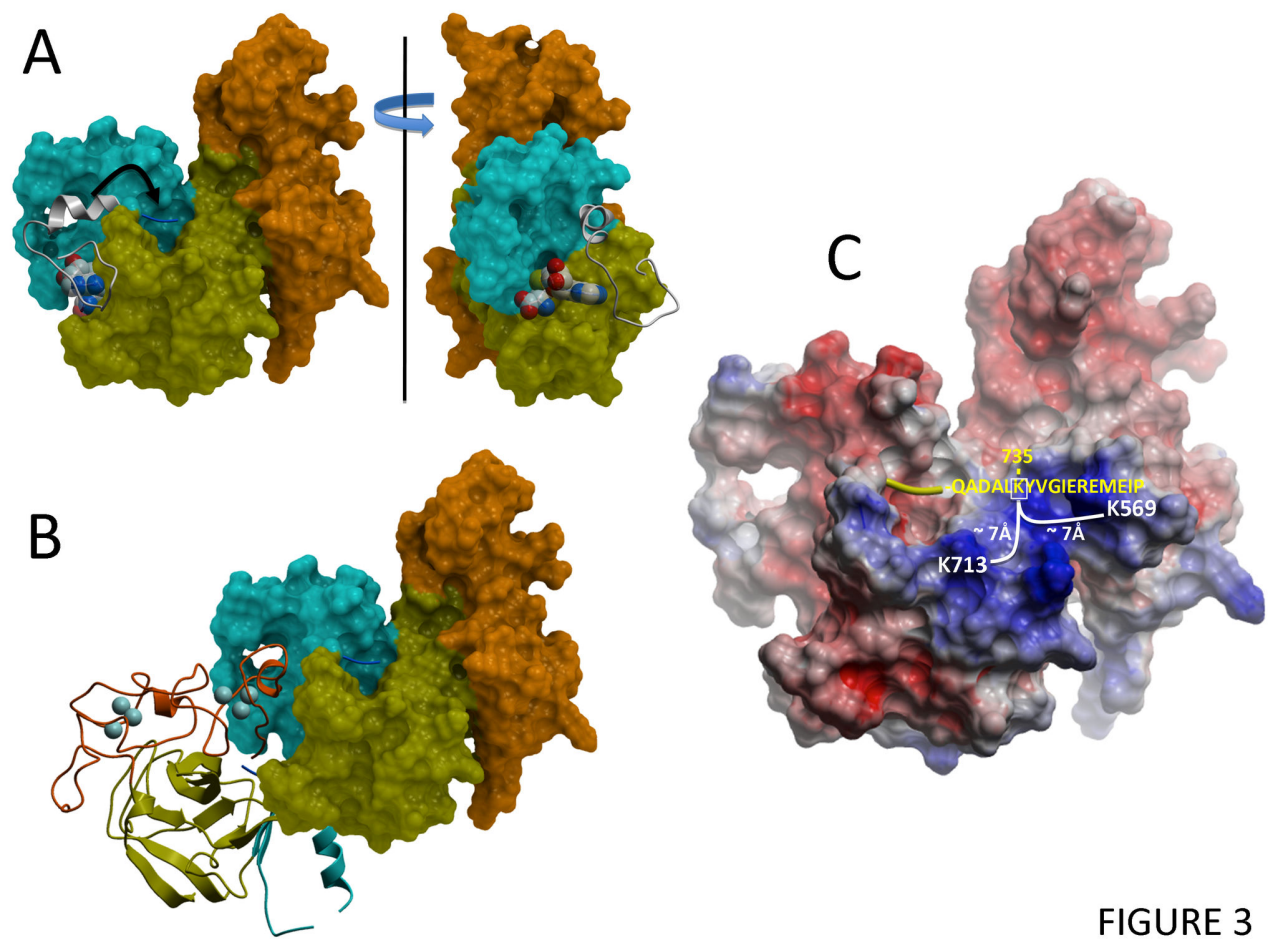
The cofactor binding site of EZH2 is incomplete. (A) Superimposition of the EZH2 structure (colored mesh; post-SET shown as blue ribbon) with a ternary complex of EHMT1/GLP (white ribbon) shows that the cofactor binding site is only partially formed in EZH2, due to an atypical orientation of the post-SET domain. (B) The cofactor site of EZH2 is occupied by the CXC domain of a second molecule within the crystal lattice. (C) Mapping of the location of lysine-mediated cross-links detected in the purified PRC2 complex [53]. Cross-links between Lys735 and Lys569 as well as Lys713 indicate that the post-SET domain of EZH2 (yellow) can project towards the CXC domain in solution, consistent with the conformation seen in our structure.

Despite the highly conserved SET domain, the EZH2 structure has several novel features (Figure 2). First, immediately N-terminal to the SET domain, a unique CXC domain, coordinated by 2 clusters of three zinc ions, differentiates EZH2 from other SET-domain PMT structures (no structural homolog of the CXC domains was found by the DALI server [40]). Missense mutations at residues coordinating the first and second zinc of the CXC domain were reported in acute myeloid leukemia (H525N) and myelodysplastic syndrome (C571N) suggesting that disruption of the CXC domain can be associated with specific cancer types [41,42]. Second, the conformation of the first five residues of the post-SET domain diverges drastically from previous structures of active SET-domain methyltransferases, and folds in a direction diametrically opposite to its expected position, where it would otherwise contribute to formation of the cofactor binding site (Figure 2 and Figure S4). Importantly, within the crystal lattice, each CXC domain interacts with a neighboring protein partially occupying both the SAM binding site and the region in which the post-SET domain is expected to reside (Figure 3). Thus, the conformation observed in our structure is not compatible with SAM binding and therefore reflects an inactive enzyme. The functional implications of both the conserved and unusual features of this structure are discussed below. Finally, we note the presence of a secondary pocket formed by the I-SET domain juxtaposed to the SAM binding site of EZH2 (Figure S5). It is unclear whether this pocket would be present in a non-crystalline environment or in the context of the PRC2 complex.

### Structural basis for altered activity of recurrent lymphoma mutations

**Figure 4:**
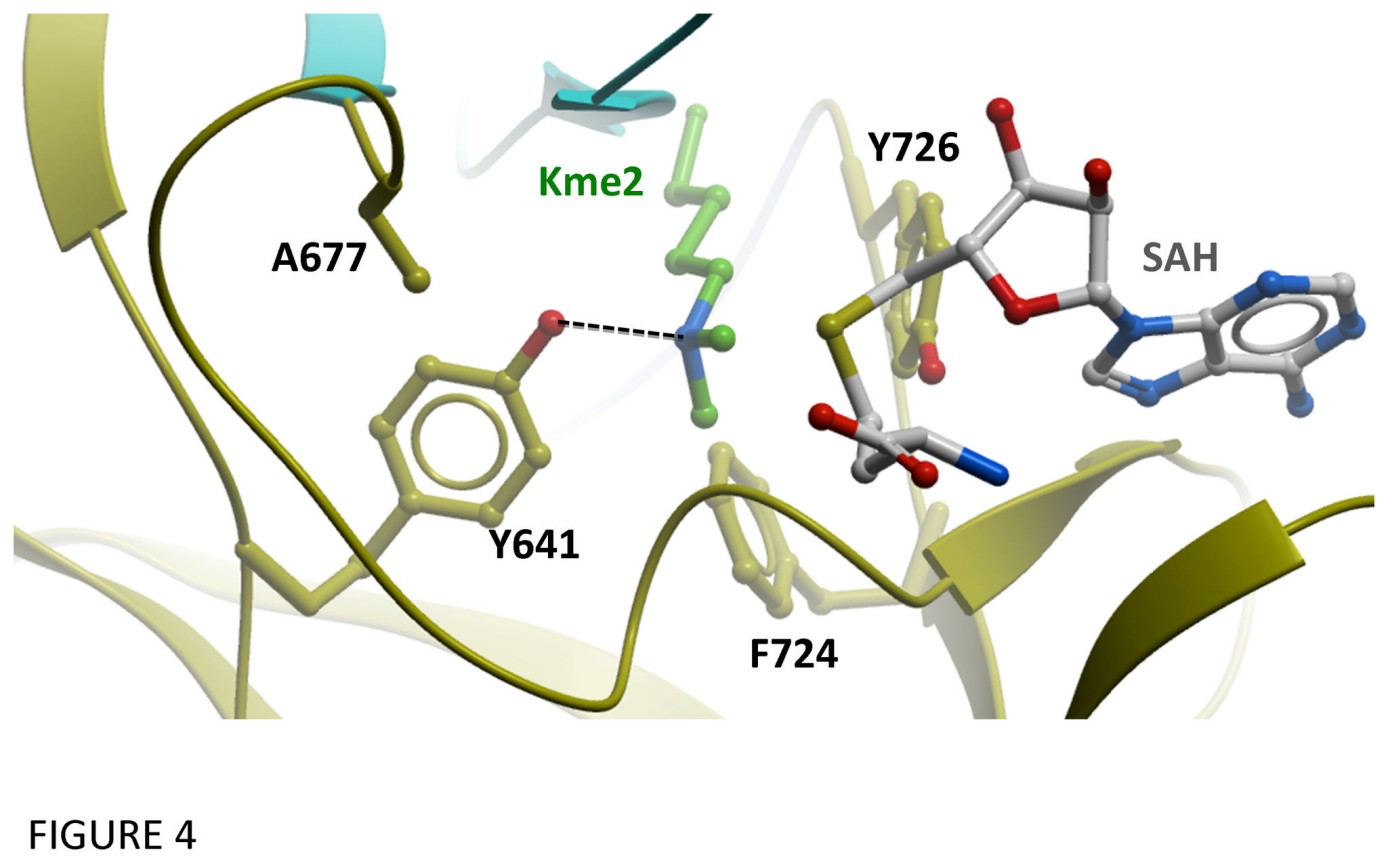
Structural basis for altered activity of mutations recurrent in lymphomas. Hydrogen bonding between Tyr 641 and the substrate lysine’s ε-nitrogen, and steric envelope of the tyrosine hydroxy group impose rotational constraints that penalize proper alignment with the cofactor’s scissile bond, required for displacement of a third methyl group. A677 stabilizes the conformation of Y641 hydrogen-bonded to the substrate lysine. The cofactor and substrate lysine are from a superimposed ternary structure of EHMT1/GLP (2RFI).

Recurrent mutations at Y641 and A677 have been shown to increase the trimethylase activity of EZH2 and drive the development of diffuse large B-cell and follicular lymphoma [21,22,23,24,25,26]. Superimposition of our EZH2 structure with that of another SET-domain methyltransferase, EHMT1/GLP, in complex with SAH and a dimethylated H3K9 peptide [43] confirms previous homology models [21,26] showing that Y641 is perfectly positioned to engage in a hydrogen-bond with the de-protonated ε-nitrogen of the substrate lysine, thereby restraining the rotational freedom of the di-methylated nitrogen atom, and disfavoring alignment of the lone pair with the scissile bond of the cofactor’s sulfonium group, necessary for the displacement of a third methyl group (Figure 4). Changing Y641 to a phenylalanine, a mutation frequently associated with lymphoma, would alleviate the conformational constraint imposed on the substrate lysine, allowing the latter to freely rotate into position for nucleophilic attack on SAM, and would provide additional space to accommodate a third methyl group. A similar mechanism was previously described for two other SET domain methyltransferases, SETD7 and EHMT2/G9a, in which mutations of the corresponding tyrosine (Y1067F in EHMT1/G9a, Y245A in SETD7) switched the predominant catalytic activity from mono- and di-methylase, respectively, to tri-methylase [43,44]. Similarly, a Tyr to Phe substitution switched SETD8 from a mono- into a di-methylase [45]. Other mutations at Y641 to asparagine, histidine and cysteine have been described that also affect the methylation specificity of EZH2 [21]. These mutations are also expected to disrupt the hydrogen-bond between the hydroxy group of the tyrosine and the substrate lysine, but otherwise be compatible with the structure of the lysine binding channel. 

Our structure also confirms a previous explanation for increased trimethylase activity of A677G mutation found in lymphoma [21]. Y641 is sandwiched between the substrate lysine and the side-chain of A677 (Figure 4). This supports an activation mechanism whereby the absence of a side chain at residue 677 allows a conformational state of Y641 that is not accessible in the wild type protein. Such an alternate confirmation could reduce the active site hydrogen-bonding potential of Y641 with the methyl-accepting nitrogen of the substrate lysine, and remove steric hindrance associated with the trimethylated state, thereby allowing proper alignment of the substrate’s dimethylated nitrogen for displacement of a methyl group from the cofactor, resulting in increased tri-methylation activity [21].

### Isolated EZH2 adopts a conformation that precludes SAM and substrate binding

**Figure 5:**
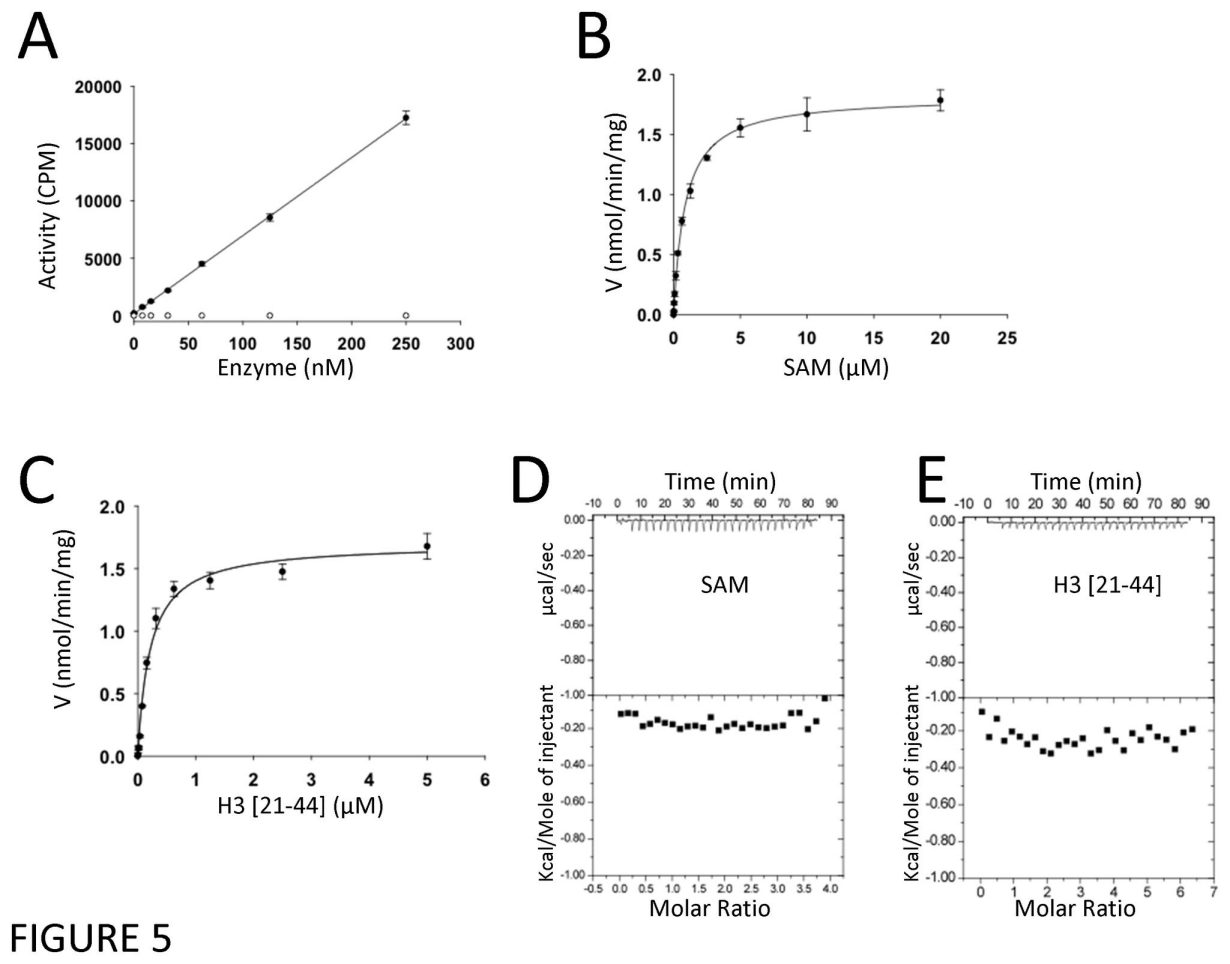
Catalytic activity and substrate/cofactor binding of EZH2 (520-746) and the trimeric (EZH2-EED-SUZ12) complex. (A) The full-length trimeric complex (●) was active, and the crystallized EZH2 construct (○) was not. Activity assay conditions were optimized for the full length EZH2 in complex with EED and SUZ12 as a control. Kinetic analysis shows that the trimeric complex binds SAM (B) and a histone peptide (C) (K_m_ SAM: 900 ± 100 nM; K_m_ peptide: 205 ± 25 nM; k_cat_: 24 ± 2 h^-1^). Apparent kinetic parameters are the average of three measurements ± standard deviation. ITC shows that the crystallized construct binds neither SAM (D) nor the peptide substrate (E).

Measurable catalytic activity for EZH2 requires the presence of PRC2 core subunits SUZ12, and EED, with incorporation of a fourth PRC2 core component, RBBP4 or RBBP7, and cofactors such as AEBP2 resulting in even greater activity [10,12,46]. We confirmed that our crystallized EZH2 construct is inactive in isolation compared to the trimeric complex of full length EZH2/EED/SUZ12 under the same conditions (Figure 5A). Since our structure indicates that the lysine-binding channel and active site appear to be competent for catalysis (Figure 1D), we tested whether the crystallized construct was capable of binding substrate or cofactor, two binding events necessary for catalysis. While kinetic analysis shows that the trimeric PRC2 complex with full-length EZH2 binds both SAM and a H3K27 peptide substrate, our crystallized EZH2 construct binds neither as measured by ITC (Figure 5D and E). This data indicates that the activity deficit in isolated, truncated EZH2 is due, at least in part, to failure to bind substrate and cofactor. There are several features of the structure, each of which can explain this lack of activity, and hint at a structural mechanism for activation of EZH2 by other PRC2 subunits. 

First, as mentioned above, the cofactor site is occupied by the CXC domain of a second EZH2 molecule (Figure 3B). Whether this feature is a crystallographic artifact or is a cause or a consequence of post-SET misfolding is unknown. However, this intermolecular interaction cannot be the sole explanation for the inactivity of EZH2 because in solution EZH2 appears to exist both as monomer and dimer (Figure S6), and we observe no catalytic activity associated with the ~50% monomeric species (Figure 5).

A second structural feature that reflects an inactive enzyme is the absence of a peptide binding groove due to the relative position of the I-SET and the first several residues of the post-SET domain. All ternary structures of SET domain methyltransferases in complex with cofactor and substrate have the substrate peptide lying in a narrow groove formed by the I-SET domain on one side, and the post-SET domain on the opposite side. This peptide binding groove is responsible for substrate sequence specificity and positions the substrate lysine side chain into a conserved channel that goes deep into the core of the SET domain and meets the cofactor at the conserved active site as described above (Figure 2) ([38,43,44,47,48,49,50,51,52] and PDB code 4AU7). The width of the peptide-binding groove is critical for catalysis, and the distance separating the two edges is between 7.2 and 7.8 Å across all available structures of active SET domain constructs (see materials and methods section for details). Southall et al have shown that in the MLL SET domain structure, the groove is more open (9.6 Å), which results in suboptimal positioning of the substrate lysine and loss of catalytic activity (Figure 2) [51]. Addition of RBBP5 and ASH2L, two other subunits of the active MLL complex, are necessary for activity and thought to contribute to completing or closing the peptide channel [51]. The EZH2 structure reveals the opposite conformational state that could have similar consequences: a shift in the I-SET domain brings it in close proximity (2.9 Å) to the first 5 residues of the post-SET domain, effectively eliminating the peptide binding groove and sealing the entrance of the substrate lysine channel. This conformation is mediated by a hydrogen bond between the backbone nitrogen of N668 in the I-SET domain and the backbone oxygen of Y726 in the post-SET (Figure 2, Figure S7). This conformational state is incompatible with binding and proper positioning of the substrate for catalysis.

A third feature that may contribute to inactivity is the position of the post-SET domain. In all other SAM or SAH-bound structures of SET domain PMTs, the post-SET domain partially contributes, along with the SET and I-SET domains, to the formation of the cofactor binding site (Figure S4). However, the post-SET domain of EZH2 appears to project towards the CXC domain, away from the expected site of cofactor recruitment, resulting in an incomplete cofactor pocket (Figure 3A). Because only 5 of 22 residues of the post-SET domain have clear electron density in our structure, the actual position of the full post-SET domain is uncertain and may have multiple conformations. However, given the trajectory of the first five residues and the small size of the post-SET domain, it is unlikely to be able to “reach” the cofactor site. Similar orientations are observed at the N-terminus of the post-SET domain in the auto-inhibited, cofactor-bound structures of SUV39H2 and SETMAR [PDB codes 2R3A, 3BO5], but the full-length post-SET of these proteins is at least 18 residues longer than that of EZH2. Furthermore, using cross-linking experiments coupled with mass spectrometry in the context of the reconstituted, purified PRC2 complex, Ciferri et al. have captured physical proximity between Lys735, in the unresolved section of the post-SET domain, and Lys569, in the CXC domain, as well as neighboring Lys713 in the SET domain [53] (Figure 3C). This suggests that the unexpected orientation of the post-SET domain observed in our structure is not a crystallographic artifact, but is at least one of several possible conformations within the PRC2 complex. 

The orientation of the EZH2 post-SET domain away from the SAM-binding site is analogous to that observed in a series of structures we previously solved for the human PRDM proteins - PRDM1 (PDB code 3DAL), PRDM2 (2QPW) [43], PRMD4 (3DB5), PRDM9 (4IJD) [54], PRDM10 (3IHX), PRDM11 (3RAY), and PRDM12 (3EP0) (Figure S8). PRDMs are methyltransferases with a core catalytic PR domain that is structurally related to the SET domain. Interestingly, the cofactor SAM is absent from all these structures, but is present in a structure of mouse PRDM9 (4C1Q) in which the post-SET domain adopts a conformation that is closer to that observed in active SET domain protein structures [54]. However, unlike EZH2, the human PRDM9 construct that crystallized in an apo, inactive conformation is nevertheless able to bind to SAM and has significant catalytic activity [54]. 

Together, these results indicate structural features underlying suboptimal binding or positioning of both cofactor and substrate in isolated EZH2, which are expected to preclude catalysis. To test whether the specific structural features observed in our structure contribute to lack of substrate or cofactor binding we designed several EZH2 mutations that would be expected to disrupt or ‘relieve’ the apparent inactive conformations of the I-SET, post-SET and CXC domains. We mutated post-SET residue Ser729 (buried due to the altered post-SET conformation) to an aspartate to destabilize the observed conformation of the post-SET domain. We also mutated I-SET residue Phe667 (engaged in orthogonal pi stacking with F724) into a leucine to remove interactions that stabilize the shifted conformation of the I-SET domain (Figure S7). Neither of these mutants, nor the double mutant was able to bind substrate or cofactor as measured by either ITC or differential scanning light scattering [55]. Attempts to prepare a construct that lacked the CXC domain (which might be expected to relieve the intermolecular interaction with, and disruption of the cofactor site) were unsuccessful due to instability of the truncated protein. 

Finally, given the recently reported potent SAM-competitive inhibitors of EZH2 [27,28,29,30,31,32] we hypothesized that their tight binding to the cofactor binding site in the PRC2 complex may confer the ability to bind to the isolated EZH2 protein, even though SAM does not bind. However, we could detect no binding for UNC1999 by ITC (data not shown), indicating that SAM competitive inhibitors also require the minimal PRC2 complex components. 

## Discussion

The crystal structure presented here reveals that EZH2 adopts a canonical SET domain methyltransferase fold in the absence of binding partners, and that the catalytic site is well formed (Figure 1D). We find that the structure of the cofactor site is compatible with the formation of four of six hydrogen bonds with the cofactor that are conserved across all SET domain complex structures (Figure S9) [56]. The structure also rationalizes the increased trimethylase activity of the mutated enzyme found in lymphomas. However, unlike the isolated SET domains of many other methyltransferases, this isolated SET domain construct of EZH2 is unable to methylate its H3K27 substrate in the absence of protein interaction partners, EED and SUZ12 [12]. Two structural features distinguish our EZH2 structure from catalytically competent conformations of other SET domain methyltransferases: the post-SET domain projects away from its expected position (Figure 3A), resulting in an incomplete cofactor binding site, and the I-SET domain is shifted towards the post-SET domain, which closes the histone binding groove and blocks the entrance of the substrate lysine channel (Figure 2 and Figure S10). While these features may be related to crystal lattice contacts which are numerous both at the post-SET and I-SET domains, there is evidence that at least some of the atypical features of our crystallized conformation (inactive trajectory of the post-SET) may be populated in the PRC2 complex in solution (Figure 3C) [53].

Our EZH2 structure contributes to the growing evidence for conformational plasticity of the I-SET and post-SET domains of SET domain methyltransferases and hints at potential mechanisms of regulation of catalytic activity. The post-SET domain participates in the formation of both cofactor and substrate binding sites, and is expected to adopt a catalytically competent conformation only when bound to both SAM and peptide ([38] for review). For instance SETD7 was captured crystallographically in apo, cofactor-bound, and cofactor- plus substrate-bound states, each with a unique conformation of the post-SET domain [44,57,58]. Similarly, (as discussed above) the related PRDM methytransferases also display extensive variability in the position of their post-SET domains [54]. Furthermore, a recent structure of SETD8 in complex with SAM (PDB code 4IJ8) compared to the ternary structure [49] reveals that structural plasticity exists also at the I-SET domain. Finally, autoinhibitory conformations where the post-SET domain occludes the substrate binding groove were reported for the H3K36 methyltransferases SETD2, SETMAR and NSD1 and the H3K9 methyltransferase SUV39H2 ([43,59,60] and PDB code 3BO5). Thus, the atypical conformations of the post-SET and (to a lesser extent) I-SET domains observed in EZH2 may therefore represent one of several conformational states available to the protein and suggest a potential mechanism for modulation of catalytic activity within the PRC2 complex. 

We propose that the other PRC2 subunits and/or N-terminal regions of full length EZH2 conspire to complete an active cofactor binding site by modulating the conformation of the post-SET and I-SET domains. Modulation of the trajectory of the post-SET domain toward the cofactor site would be expected to ‘release’ the I-SET from interaction with the post-SET thereby opening up the peptide-binding groove for substrate. Alternatively, interactions of PRC2 components with the I-SET may reposition it into a conformation more consistent with other active SET domain proteins, thereby releasing the post-SET to adopt an active conformation. Finally, it is entirely possible that a complex and possibly dynamic series of protein-protein and inter-domain interactions take place within the functional PRC2 complex to carry out its catalytic function.

## Supporting Information

Figure S1 **Electron density map.** Sample electron density map of the substrate lysine channel.
(TIF)

Figure S2 **EZH2, EHMT1 and vSET catalytic sites.** The catalytic site of EZH2 is structurally closer to that of the human H3K9 dimethylase EHMT1 than the viral H3K27 trimethylase vSET .
(TIF)

Figure S3 **Electrostatic potential.** The expected location of the substrate peptide binding site of EZH2, at the interface of the post-SET (blue) and I-SET (cyan) domains, is electronegative. Bottom: electrostatic potential color coding. Blue: electropositive; red: electronegative.
(TIF)

Figure S4 **Post-SET domain conformation.** The post-SET domain (blue) of cofactor-bound SET domain methyltransferases is structurally diverse but always participates in the formation of the cofactor site. In the EZH2 structure, it projects away from its expected position and the cofactor is absent. When present, cofactor is shown as CPK and substrate is in green.
(TIF)

Figure S5 **EZH2’s secondary pocket.** A mesh representation of EZH2 (color-coding as in other figures) with the cofactor of a superimposed EHMT1/GLP structure (conserved hydrogen-bonds are highlighted), reveals the existence of a secondary pocket, juxtaposed to the cofactor site.
(TIF)

Figure S6 **EZH2’s dimeric state in solution.** EZH2 elutes both as a monomer and dimer out of a gel filtration column.
(TIF)

Figure S7 **Interactions between the post-SET and I-SET domains.** The altered orientation of the post-SET domain, resulting in incomplete formation of the cofactor site, is associated with a buried conformation of Ser 729. The shifted orientation of the I-SET domain, resulting in closure of the substrate-binding groove, is stabilized by a hydrogen-bond between the backbone of N668 and Y726, and orthogonal pi-stacking between Phe 667 and Phe 724. Color coding as in other figures.
(TIF)

Figure S8 **Post-SET domain in PRDM structures.** The post-SET domain in all human PRDM structures (blue) is oriented away from the putative cofactor site, and the cofactor is absent from all these structures. In a mouse PRDM9 structure crystallized in complex with SAH (green sticks), the post-SET domain (green ribbon) is folded on the cofactor. Mesh representation of human PRDM9 where the post-SET domain was truncated. Post-SET domain of human PRDM1 (PDB code 3DAL), PRDM2 (2QPW Wu 20084102), PRMD4 (3DB5), PRDM9 (4IJD), PRDM10 (3IHX), PRDM11 (3RAY), and PRDM12 (3EP0), and mouse PRDM9 (4C1Q).
(TIF)

Figure S9 **Conserved, but incomplete folding of the cofactor-binding site.** The cofactor site of EZH2 is in a conformational state that is compatible with the formation of 4 out of 6 hydrogen bonds (black) between the SET domain and the cofactor that are conserved across all available structures of cofactor-bound SET-domain methyltransferases. Preserved hydrogen bonds are shown in cyan. Lost hydrogen bonds are shown in magenta. The EZH2 structure (color coding as in other figures) is superimposed with cofactor-bound EHMT1/GLP (beige - PDB code 2RFI). Top-right: same view, with a mesh representation of EZH2, where the EHMT1/GLP ribbon was removed.
(TIF)

Figure S10 **Atypical conformations of the I-SET and post-SET domains.** Superimposition of the EZH2 structure (I-SET domain: cyan; post-SET domain: blue) with ternary complexes of EHMT1/GLP (PDB code 2RFI), SETD7 (PDB code 1O9S) and SETD8 (PDB code 1ZKK) bound to cofactor (balls and sticks) and substrate (no shown) shows that the I-SET domain of EZH2 is shifted towards the post-SET domain, resulting in hydrogen-bonding between Asn 668 and Tyr 726.
(TIF)
