# Supplementary figures and images for: Structure of the Catalytic Domain of EZH2 Reveals Conformational Plasticity in Cofactor and Substrate Binding Sites and Explains Oncogenic Mutations
Source: PLoS One. 2013 Dec 19;8(12):e83737. doi: 10.1371/journal.pone.0083737 (PMC3868588; doi:10.1371/journal.pone.0083737)

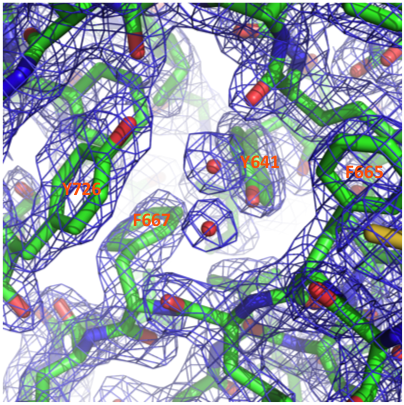

Supplement: Figure S1 — Electron density map. Sample electron density map of the substrate lysine channel. (TIF) [file pone.0083737.s001.tif]

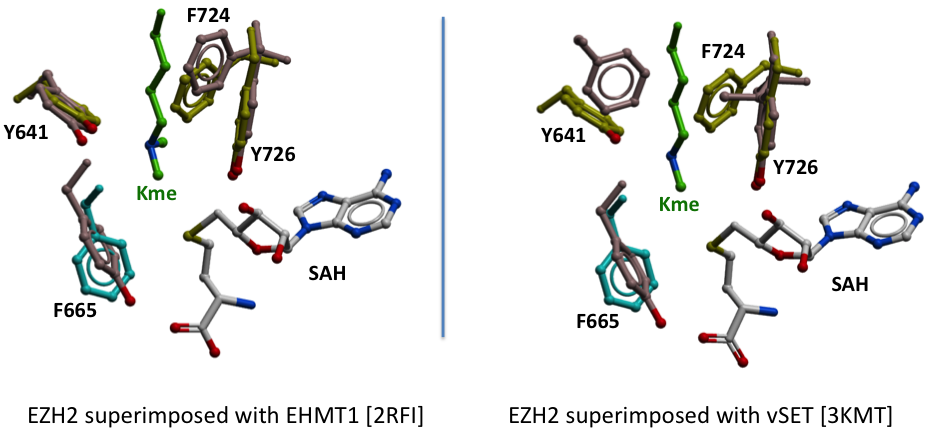

Supplement: Figure S2 — EZH2, EHMT1 and vSET catalytic sites. The catalytic site of EZH2 is structurally closer to that of the human H3K9 dimethylase EHMT1 than the viral H3K27 trimethylase vSET . (TIF) [file pone.0083737.s002.tif]

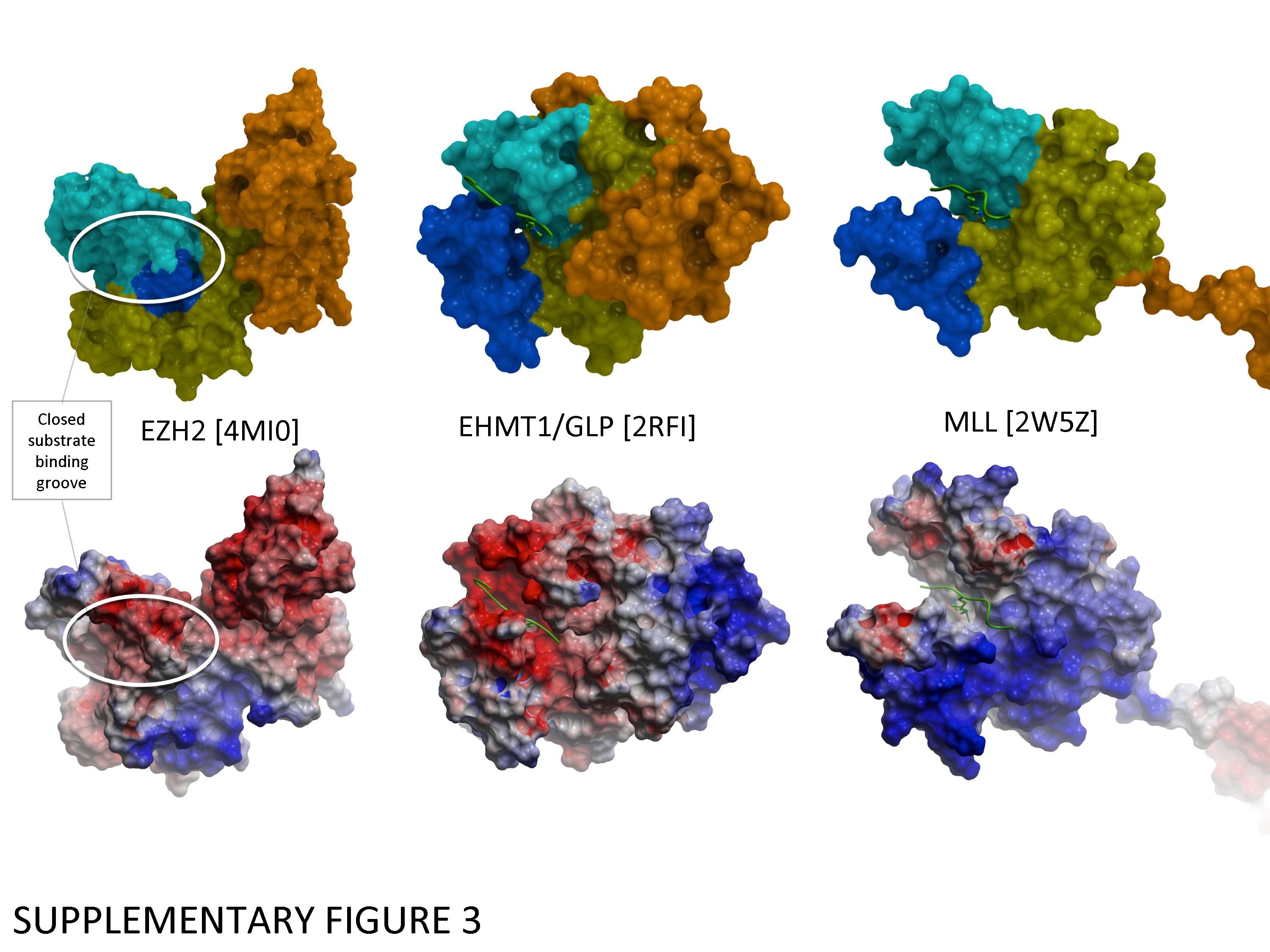

Supplement: Figure S3 — Electrostatic potential. The expected location of the substrate peptide binding site of EZH2, at the interface of the post-SET (blue) and I-SET (cyan) domains, is electronegative. Bottom: electrostatic potential color coding. Blue: electropositive; red: electronegative. (TIF) [file pone.0083737.s003.tif]

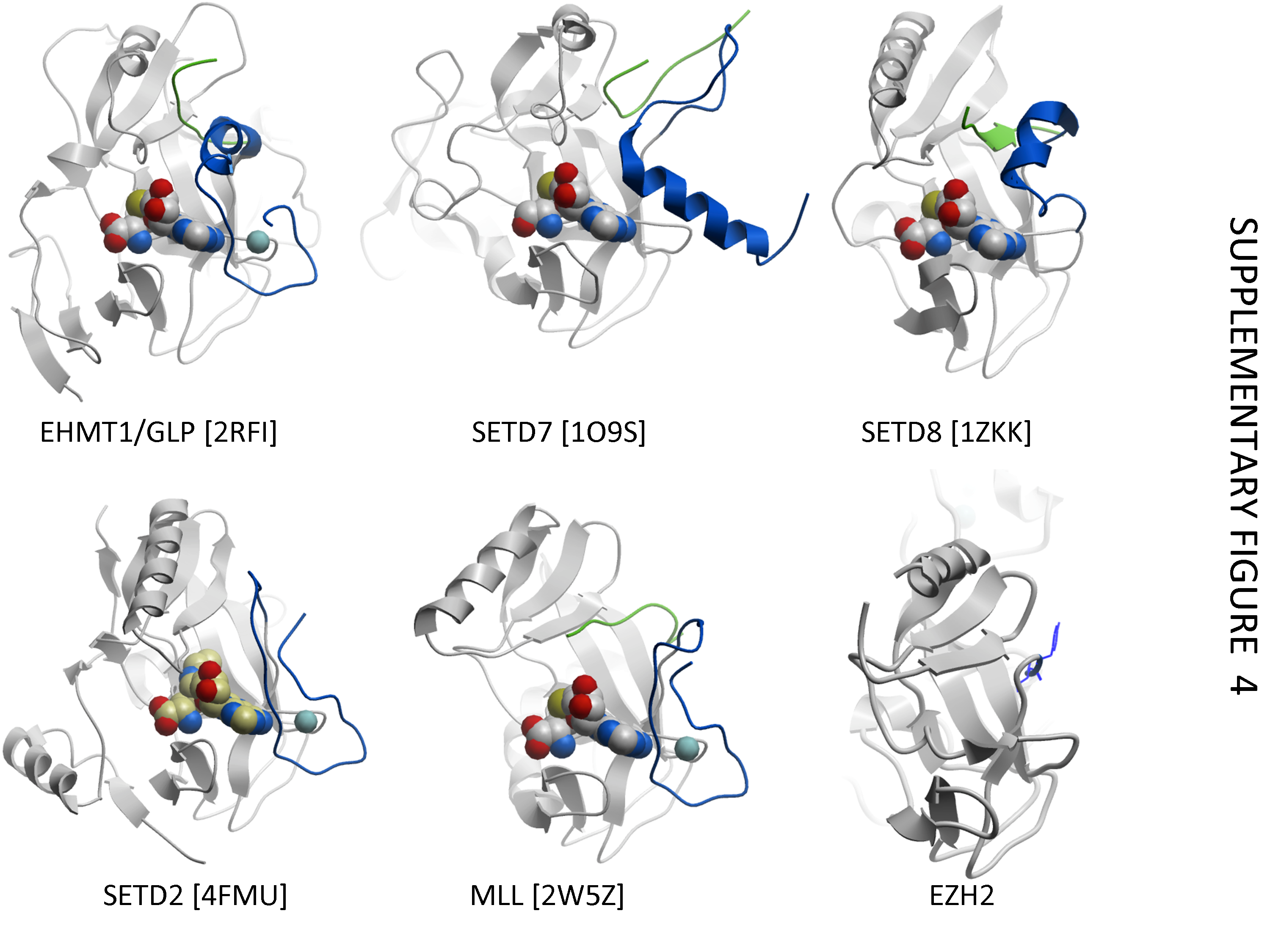

Supplement: Figure S4 — Post-SET domain conformation. The post-SET domain (blue) of cofactor-bound SET domain methyltransferases is structurally diverse but always participates in the formation of the cofactor site. In the EZH2 structure, it projects away from its expected position and the cofactor is absent. When present, cofactor is shown as CPK and substrate is in green. (TIF) [file pone.0083737.s004.tif]

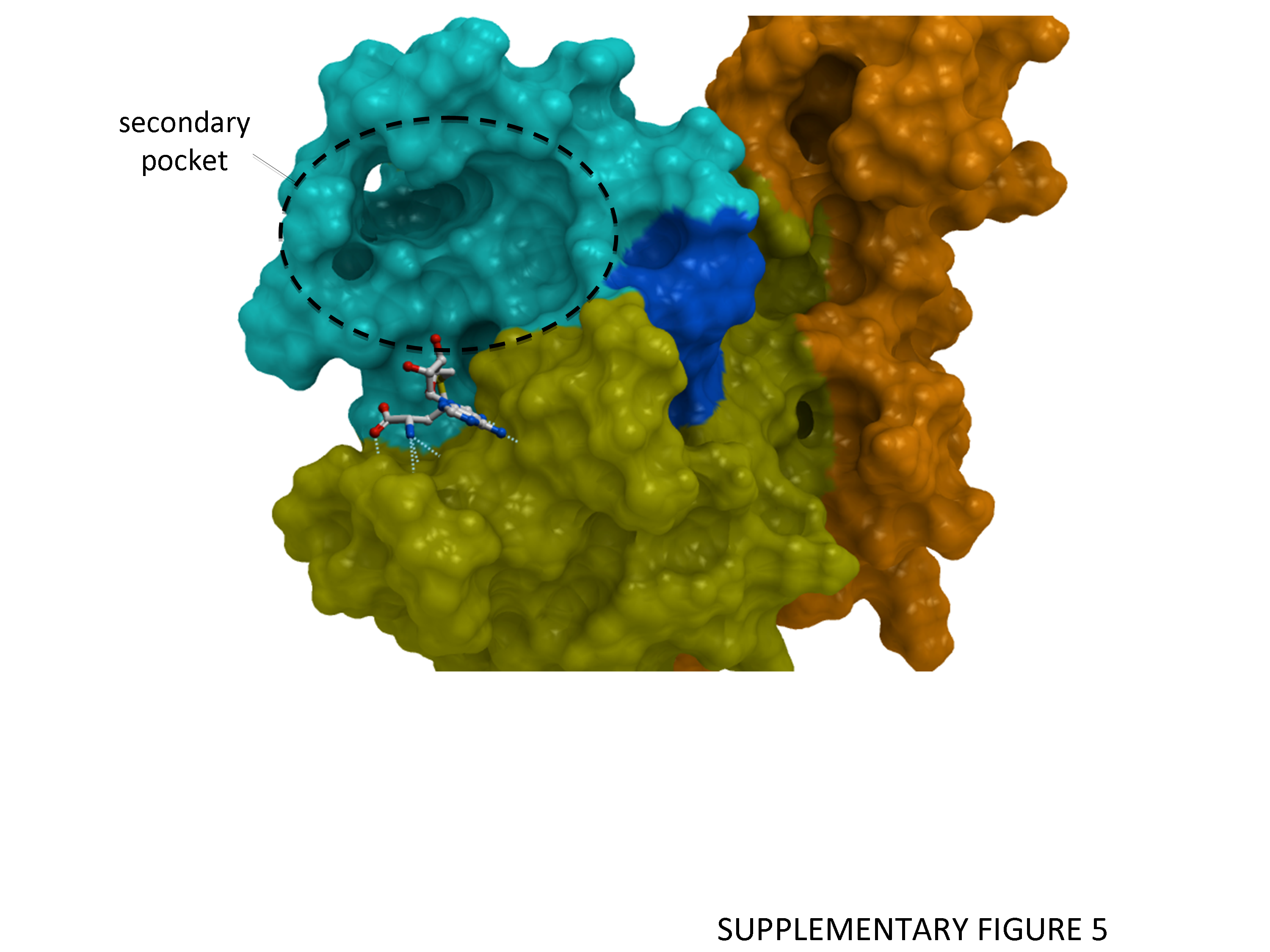

Supplement: Figure S5 — EZH2’s secondary pocket. A mesh representation of EZH2 (color-coding as in other figures) with the cofactor of a superimposed EHMT1/GLP structure (conserved hydrogen-bonds are highlighted), reveals the existence of a secondary pocket, juxtaposed to the cofactor site. (TIF) [file pone.0083737.s005.tif]

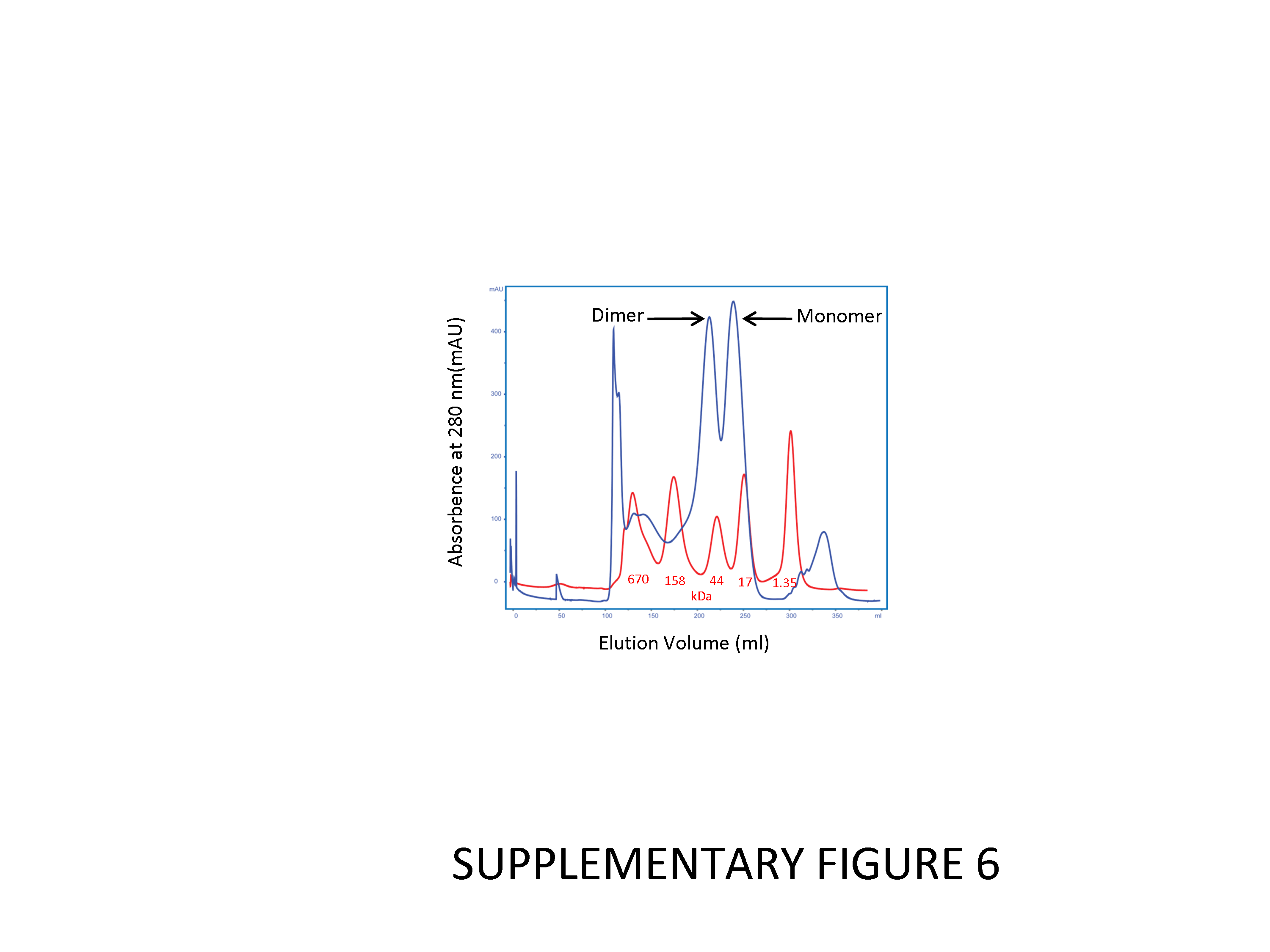

Supplement: Figure S6 — EZH2’s dimeric state in solution. EZH2 elutes both as a monomer and dimer out of a gel filtration column. (TIF) [file pone.0083737.s006.tif]

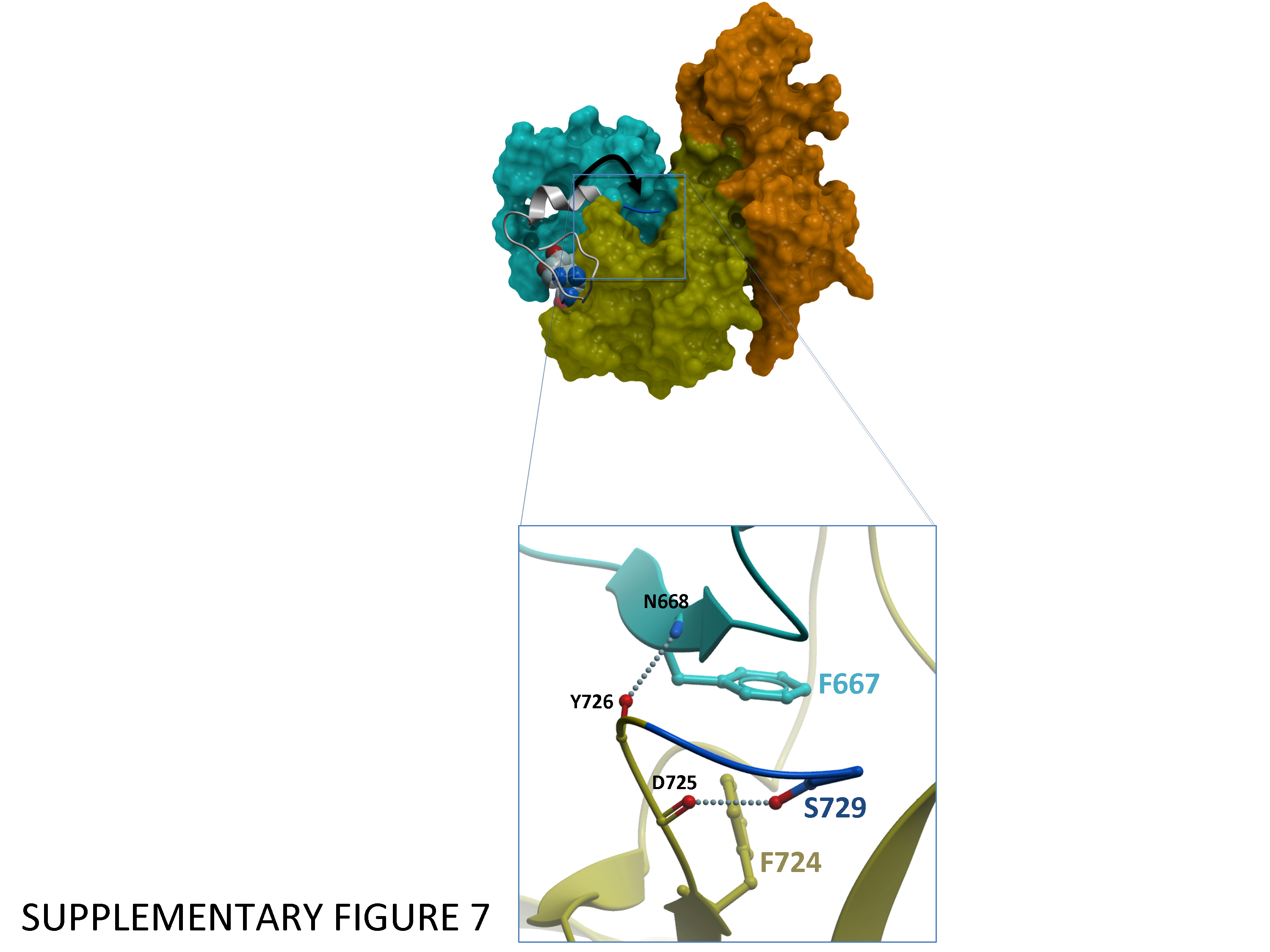

Supplement: Figure S7 — Interactions between the post-SET and I-SET domains. The altered orientation of the post-SET domain, resulting in incomplete formation of the cofactor site, is associated with a buried conformation of Ser 729. The shifted orientation of the I-SET domain, resulting in closure of the substrate-binding groove, is stabilized by a hydrogen-bond between the backbone of N668 and Y726, and orthogonal pi-stacking between Phe 667 and Phe 724. Color coding as in other figures. (TIF) [file pone.0083737.s007.tif]

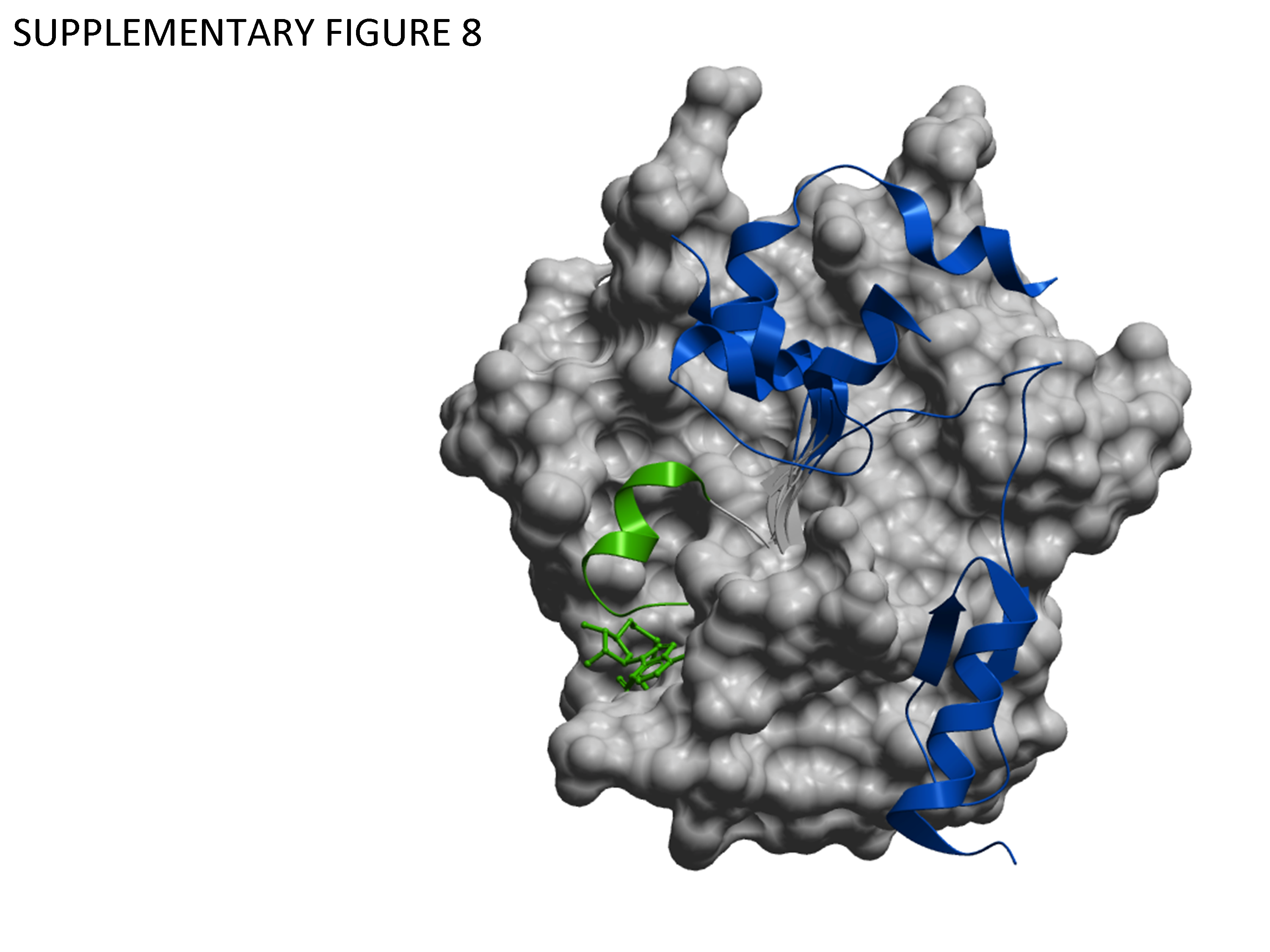

Supplement: Figure S8 — Post-SET domain in PRDM structures. The post-SET domain in all human PRDM structures (blue) is oriented away from the putative cofactor site, and the cofactor is absent from all these structures. In a mouse PRDM9 structure crystallized in complex with SAH (green sticks), the post-SET domain (green ribbon) is folded on the cofactor. Mesh representation of human PRDM9 where the post-SET domain was truncated. Post-SET domain of human PRDM1 (PDB code 3DAL), PRDM2 (2QPW Wu 20084102), PRMD4 (3DB5), PRDM9 (4IJD), PRDM10 (3IHX), PRDM11 (3RAY), and PRDM12 (3EP0), and mouse PRDM9 (4C1Q). (TIF) [file pone.0083737.s008.tif]

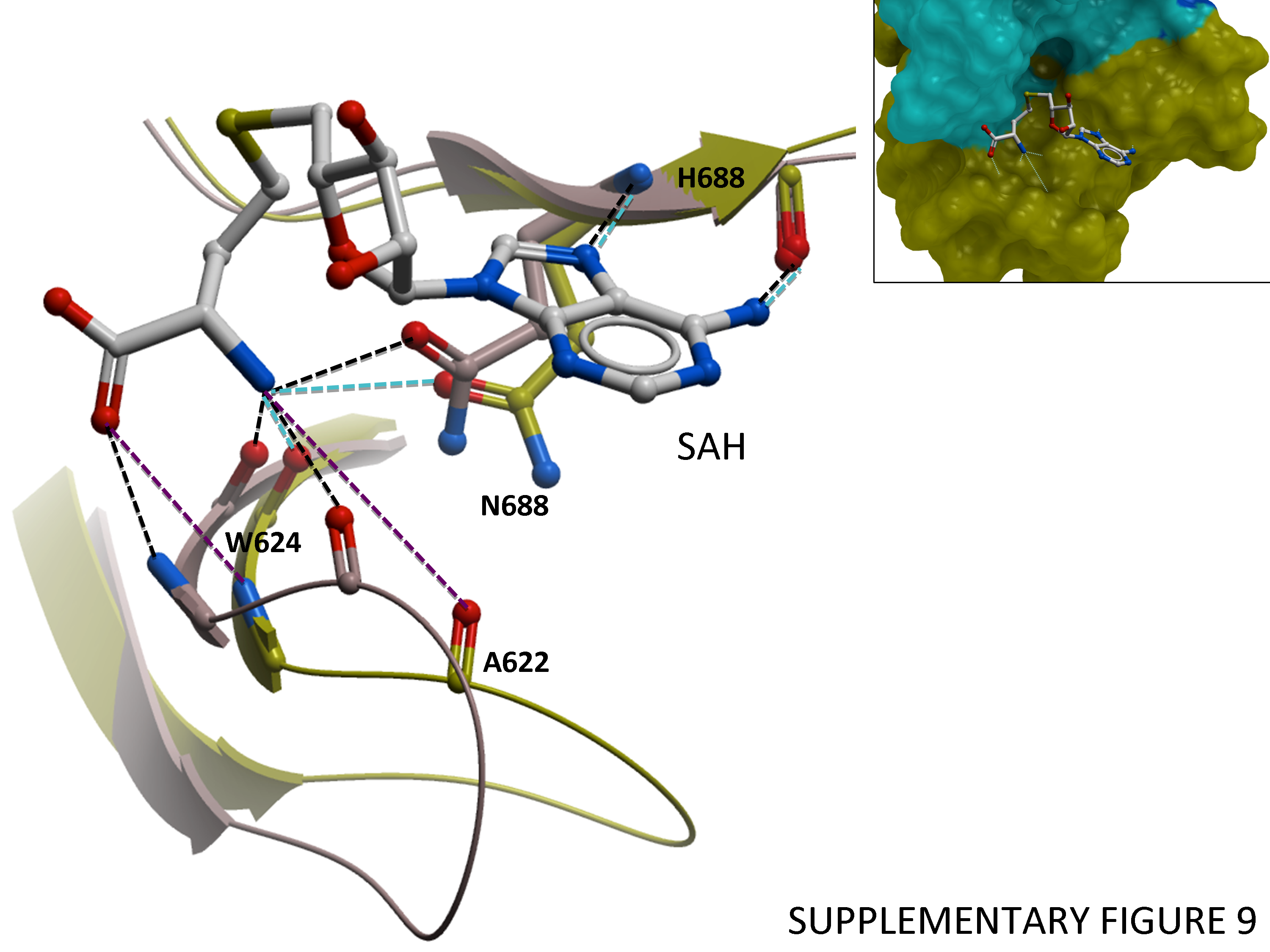

Supplement: Figure S9 — Conserved, but incomplete folding of the cofactor-binding site. The cofactor site of EZH2 is in a conformational state that is compatible with the formation of 4 out of 6 hydrogen bonds (black) between the SET domain and the cofactor that are conserved across all available structures of cofactor-bound SET-domain methyltransferases. Preserved hydrogen bonds are shown in cyan. Lost hydrogen bonds are shown in magenta. The EZH2 structure (color coding as in other figures) is superimposed with cofactor-bound EHMT1/GLP (beige - PDB code 2RFI). Top-right: same view, with a mesh representation of EZH2, where the EHMT1/GLP ribbon was removed. (TIF) [file pone.0083737.s009.tif]

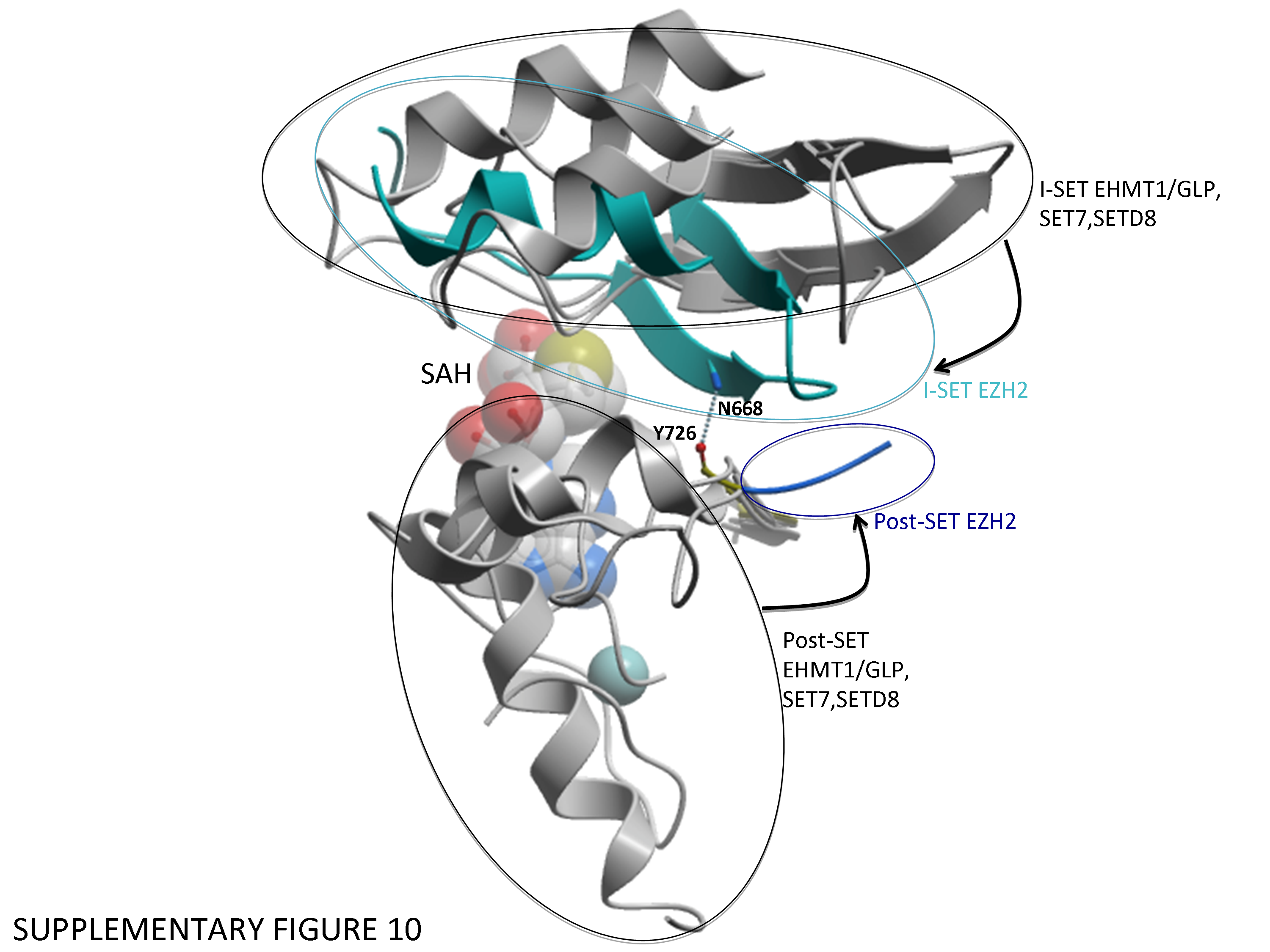

Supplement: Figure S10 — Atypical conformations of the I-SET and post-SET domains. Superimposition of the EZH2 structure (I-SET domain: cyan; post-SET domain: blue) with ternary complexes of EHMT1/GLP (PDB code 2RFI), SETD7 (PDB code 1O9S) and SETD8 (PDB code 1ZKK) bound to cofactor (balls and sticks) and substrate (no shown) shows that the I-SET domain of EZH2 is shifted towards the post-SET domain, resulting in hydrogen-bonding between Asn 668 and Tyr 726. (TIF) [file pone.0083737.s010.tif]
